# Dataset of ptychographic X-ray computed tomography of inverse opal photonic crystals produced by atomic layer deposition

**DOI:** 10.1016/j.dib.2018.10.076

**Published:** 2018-10-27

**Authors:** Kaline P. Furlan, Emanuel Larsson, Ana Diaz, Mirko Holler, Tobias Krekeler, Martin Ritter, Alexander Yu. Petrov, Manfred Eich, Robert Blick, Gerold A. Schneider, Imke Greving, Robert Zierold, Rolf Janßen

**Affiliations:** a Institute of Advanced Ceramics, Hamburg University of Technology, Denickestraße 15, 21073 Hamburg, Germany; b Center for Hybrid Nanostructures, Universität Hamburg, Luruper Chaussee 149, 22607 Hamburg, Germany; c Institute of Materials Research, Helmholtz-Zentrum Geesthacht, Max-Planck-Strasse 1, 21502 Geesthacht, Germany; d Paul Scherrer Institut, 5232 Villigen PSI, Switzerland; e Electron Microscopy Unit, Hamburg University of Technology, Eißendorfer Straße 42, 21073 Hamburg, Germany; f Institute of Optical and Electronic Materials, Hamburg University of Technology, Eißendorfer Straße 38, 21073 Hamburg, Germany; g ITMO University, 49 Kronverkskii Avenue, 197101 St. Petersburg, Russia

## Abstract

This data article describes the detailed parameters for synthesizing mullite inverse opal photonic crystals via Atomic Layer Deposition (ALD), as well as the detailed image analysis routine used to interpret the data obtained by the measurement of such photonic crystals, before and after the heat treatment, via Ptychographic X-ray Computed Tomography (PXCT). The data presented in this article are related to the research article by Furlan and co-authors entitled “Photonic materials for high-temperature applications: Synthesis and characterization by X-ray ptychographic tomography” (Furlan et al., 2018). The data include detailed information about the ALD super-cycle process to generate the ternary oxides inside a photonic crystal template, the raw data from supporting characterization techniques, as well as the full dataset obtained from PXCT. All the data herein described is publicly available in a Mendeley Data archive “Dataset of synthesis and characterization by PXCT of ALD-based mullite inverse opal photonic crystals” located at https://data.mendeley.com/datasets/zn49dsk7x6/1 for any academic, educational, or research purposes.

**Specifications table**

**Table:** 

Subject area	Materials Science
More specific subject area	*Ceramics and Composite; Nanotechnology; Surfaces, Coatings and Films*
Type of data	*Table, image, text file, graph, figure*
How data was acquired	a. *Scanning electron microscopy (SEM, Zeiss Supra 55 VP)*; b. *Focused ion beam microscopy (FIB, FEI Helios Nanolab G3 UC)*; c. *Ptychographic X-ray computed tomography (cSAXS beamline - Swiss Light Source, Paul Scherrer Institut, 90 K, 6.2 keV photon energy, 800 projections, 0–180° angle)* d. *3D rendering images (2D reconstructed and processed slices were rendered using VG Studio MAX Software, Volume Graphics)* e. *Grazing incidence X-ray diffraction (Bruker AXS D8 Advance, Cu Kα, 40 kV, 40 mA, step size 0.01°, step time 5 s, incident glancing angle 1.5°)*; f. *Specular reflectance (UV–vis-NIR spectrometer, Perkin-Elmer, Lambda 1050).*
Data format	Raw: Ptychographic X-ray Computed Tomography slices (*.TIF); Reflectance measurements (*.SP-datei, *.CSV); X-ray diffraction (*.RAW); SEM (*.TIF)
	Processed: Ptychographic X-ray Computed Tomography data – Re-sliced slices (*.TIF)
	Analyzed: text file, graphs and figures (*.TIF and *.DOCX)
	True metadata: text files (*.TXT) containing information about the the pixel size in the tomography raw-slices, and the conversion from tiff values to refractive index and electron density
Experimental factors	*Samples, Self-assembly conditions, Atomic layer deposition process parameters, heat treatment conditions*
Experimental features	*Parameters for the synthesis of oxide-based ceramic inverse opal photonic crystals by Atomic Layer Deposition and characterization of the synthesized specimens by Ptychographic X-ray Computed Tomography comprising an post-measurement image analysis routine*
Data source location	*Hamburg, Germany, latitude 53.4603824 longitude 9.96856071271512; Hamburg, Germany, latitude 53.57492275 longitude 9.885808585389755; Villigen, Switzerland, latitude 47.5375329 longitude 8.2224404*
Data accessibility	*Data are presented in this article and publicly available via Mendeley Data at* https://data.mendeley.com/datasets/zn49dsk7x6/draft?a=bb48c4e0-8fa7-4e9d-9028–754735b37d1f http://dx.doi.org/10.17632/zn49dsk7x6.1
Related research article	[1]*K.P. Furlan, E. Larsson, A. Diaz, M. Holler, T. Krekeler, M. Ritter, A.Yu. Petrov, M. Eich, G.A. Schneider, R. Blick, I. Greving, R. Zierold, R. Janssen, Photonic materials for high-temperature applications: synthesis and characterization by X-ray ptychographic tomography, Applied Materials Today (2018) [in press].*

**Value of the data**

• Detailed description of the atomic layer deposition (ALD) super-cycle process might be used to deposit mullite in different templates/substrates other than the one studied in Furlan et al. [1].
• Detailed image analysis routine description might be used for interpretation of other datasets obtained either by Ptychographic X-ray Computed Tomography (PXCT) or nanotomography of highly-porous samples, such as, but not limited to, inverse opal photonic crystals, hollow-core photonic glasses, nano membranes, or solid oxide fuel cells.
• Detailed image analysis routine description might be used for processing raw data obtained from PXCT or nanotomography measurements.
• Reconstructed slices from PXCT measurements might be used as input for sintering simulations or optical properties modelling.

## 1 Data

The data comprise detailed information about the atomic layer deposition super-cycle processes to infiltrate polymeric templates with mullite (ceramic ternary oxide), as well as the characterization of the obtained inverse opal photonic crystals via Ptychographic X-ray Computed Tomography (PXCT). Moreover, it describes in detail the image analysis routine used to interpret the data obtained by the PXCT. The data include the PXCT reconstructed and processed slices, as well as the raw data for other characterizations techniques (SEM, XRD, and specular reflectance) used by Furlan et al. in [1].

## 2 Experimental design, materials, and methods

### 2.1 Low-temperature atomic layer deposition super-cycle process

**Fig. 1:**
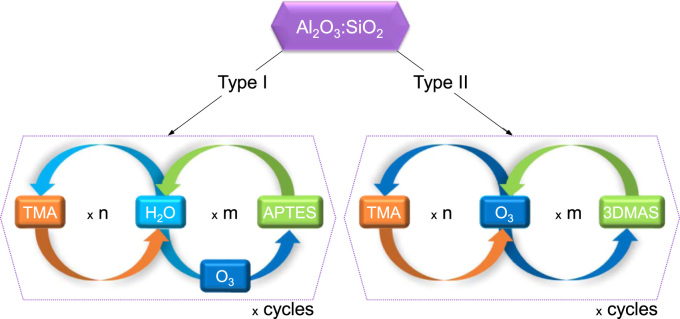
Schematic drawing of the low-temperature ALD super-cycles approach used for infiltration of photonic crystals with mullite.

The ratio of Al_2_O_3_:SiO_2_ was estimated by using the individual compound growth per cycle (GPC) and reported films densities [2], [3], [4], and varied by the number of internal loops within the super-cycle (Table 1). A schematic drawing of this process is shown in Fig. 1.

The binary process of each individual loop was developed by deposition of films onto silicon wafers (as received, <100>, native oxide layer, Si-Mat silicon materials), followed by thickness and refractive index measurements by spectroscopic ellipsometry (SENProTM, SENTECH Instruments GmbH) with a halogen lamp and a 70° incident angle.

After binary processes refinement, the super-cycles development was performed first by depositions made onto silicon wafers and later by infiltration of polystyrene templates generated by self-assembly. Once the full homogeneous infiltration of the polystyrene templates was achieved and inverse opal photonic crystals were successfully obtained after burn-out of the PS template, the recipe for mullite was recorded. Deposition was also performed onto BaF_2_ wafers (as received, <111>, Crystal GmbH) to measure the chemical composition of the deposited films by EDX (not possible on the Si wafers or sapphire substrates due to the presence of Si, Al, and O atoms, which would invalidate the composition quantification). The precursors used for the depositions were deionized water (diH_2_O), trimethylaluminum, min. 98% (TMA, Strem Chemicals), (3-aminopropyl)triethoxysilane, 98% (APTES, Sigma-Aldrich), Ozone (OzoneLabTM, OL80W), and Tris(dimethylamino)silane, 99+% (TDMAS, Strem Chemicals). While TMA was kept at room temperature, diH2O, APTES, and TDMAS were heated up to 40 °C, 95 °C, and 40 °C, respectively. A summary of the precursors and parameters used for all deposition processes are described in Table 1.

**Table 1:** Parameters of ALD cycles.

Process		Precursors used	Parameters*	Cycles
#	Description			
1	Al_2_O_3_[5]	TMA + diH_2_O	0.2/60/90 + 0.05/60/90	300
2	SiO_2_ type II [6]	APTES + diH_2_O + O_3_	2/15/45 + 0.2/15/45 + 0.05/10/30	600
3	SiO_2_ type I [7], [8], [9], [10]	TDMAS + O_3_	0.05/30/30 + 0.05/10/30	300
4	Mullite type I (combination between binary cycles #1 and #3)	[(3DMAS + O_3_) × 1+ (TMA + O_3_) × 1) × Cycles	(0.05/60/90 + 0.05/60/90) + (0.2/60/90 + 0.05/60/90)	225
3	Mullite type II (combination between binary cycles #1 and #2)	[(APTES + diH_2_O + O_3_) × 2 + (TMA + O_3_) × 1] × Cycles	(2/30/120 + 0.2/30/360 + 0.05/30/90) + (0.2/30/90 + 0.05/30/120)	150

* Pulse/exposure/pump time (s).

### 2.2 PXCT samples’ preparation

**Fig. 2:**
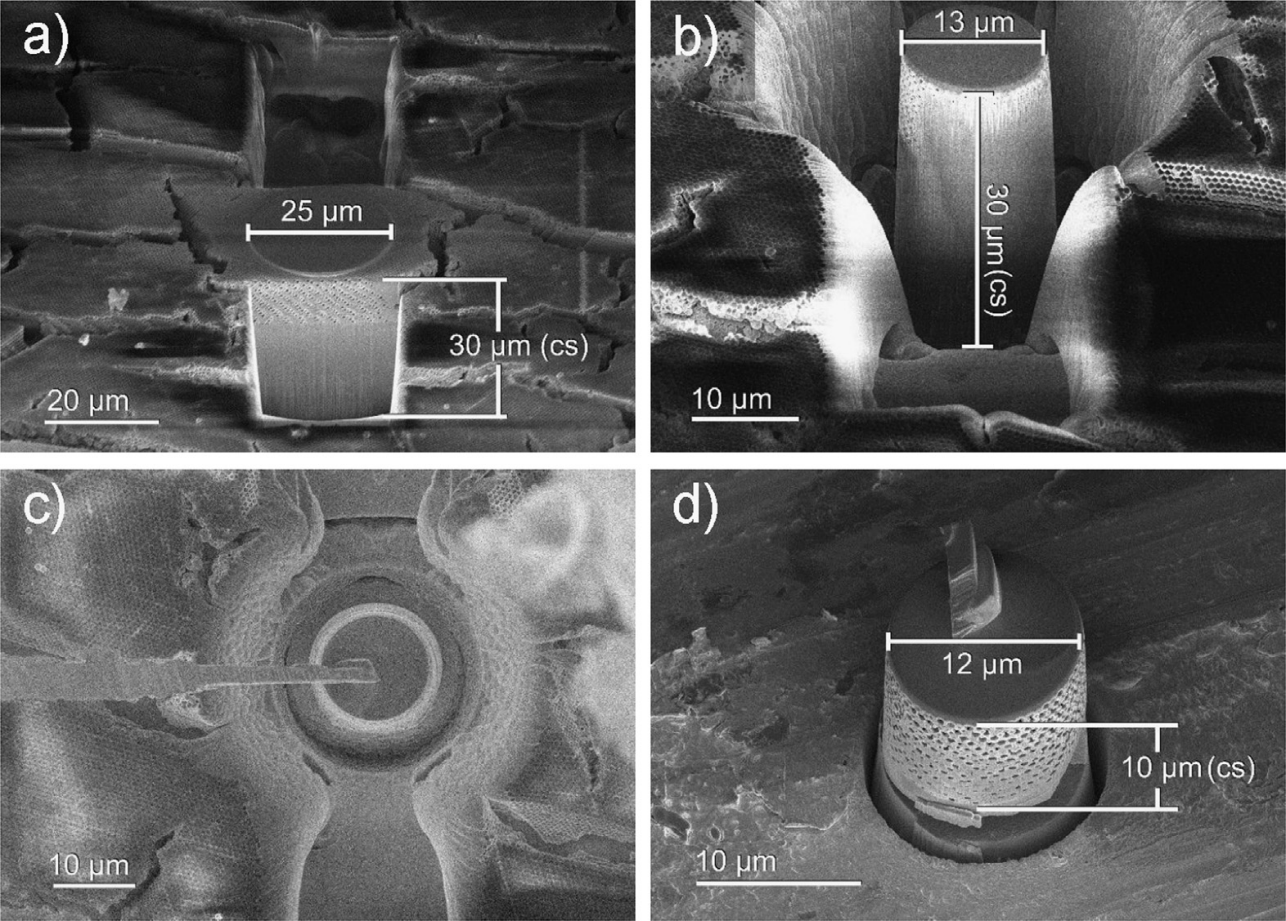
Details of samples preparation steps for PXCT measurements. (a) Pt protected sample area exposed for annular milling and lift out (stage tilt 52°), (b) specimen after annular milling down to 15 µm with 9.3 nA (stage tilt 52°), and (c) sample lift out. Tungsten needle welded to Pt layer (stage tilt 0°) and (d) sample placed onto ptychographic-specific sample holder after final polishing step (stage tilt 40°).

As both the inverse opal photonic crystal structures and the sapphire substrate are prone to charging, the entire substrate was sputtered with gold before transfer to the FIB. To prevent damage of the delicate inverse opal photonic crystal structure, a thin layer of platinum was deposited by electron beam deposition (30 kV/6.4 nA). Afterwards a 1 µm Pt protective layer was deposited with Ga+ at 30 kV and a current density of 5 pA/µm² as a filled circular pattern of 25 µm diameter (see Fig. 2a).

The area for the pillar shaped specimen was exposed by two large cross-sectioning patterns with a beam current of 21 nA (Fig. 2a) in front of and behind the circular Pt pattern. The pillar itself was prepared by a series of annular milling steps with decreasing inner diameter and beam current from 22 to 15 µm and 21 to 9.3 nA, respectively (Fig. 2b). The pillar was extracted at a stage tilt of 0° by means of an in situ Lift-out needle after cutting the pillar right below the region of interest with a current of 9.3 nA (Fig. 2c). To increase the contact area to the ptychography sample holder [11], the inclined stem of the pillar was flattened by Ga+ milling before welding it to the sample holder by platinum deposition. The final FIB-polishing step of the pillar to a diameter of 12 µm was performed by annular milling with a beam current of 0.8 nA (Fig. 2d).

The two samples prepared by FIB were milled out of one former sample, that is, the sample was the same up to the burn-out of the polymeric template, when it was then sectioned for the heat treatment. This was done to reduce the influence of the processing parameters in the sample, namely from the vertical convective self-assembly and ALD super=cycle, thus allowing us to compare PXCT data acquired from both samples. Thereby, we consider that the changes in the structure, described and explained in the PXCT analysis presented in [1], are caused solely the sintering of the structure/effect of the heat treatment, not from the self-assembly (which shall define the period by the polymeric particles packing) or ALD (which could slight alter the period), as the processing parameters up to heat treatment were exactly the same. For a clear definition and explanation of ‘period’, refer to Fig. 8 in [1] and the manuscript text related to it.

### 2.3 PXCT measurements

The OMNY instrument described by Holler et al*.* [12] was used to perform the PXCT measurements. A 220 µm diameter gold Fresnel zone plate (FZP) in combination with a 50 µm central stop and 30 µm diameter order sorting aperture were used to define the beam on the sample. The focal distance of the FZP was 66 mm and the sample was placed at 1.2 mm after the focus resulting in a beam size of 4 µm on the sample. Coherent diffraction patterns were recorded using a Pilatus 2 M detector positioned at a distance of 7306 mm after the sample. The scanning positions followed a Fermat spiral trajectory [13] with a step size of 1.2 µm and an exposure time of 0.1 s per point. Projections were reconstructed from 400 × 400 pixels of the detector using 300 iterations of the difference map algorithm [14] followed by 400 iterations of a maximum likelihood optimization [15], resulting in a real space pixel size of 21.2 nm. Reconstructed phase images were further processed to remove zero and linear terms and subpixel registration before tomographic reconstruction by filtered back projection, as described in [16], [17].

### 2.4 Image post processing and analysis

The reconstructed slices were down-converted to a 8-bit using the ImageJ software, Fiji distribution [18], to allow further quantitative analysis using the Pore3D software package [19]. A 3D mean filter (size: 3 × 3 × 3 pixels^3^) implemented in Image J [20] was applied to remove image noise and facilitate further image segmentation. Image segmentation of pores and photonic crystal phases was performed using an automated (supervised) pixel-level classification method (based on intensity, edge and texture with sigma = 0.7) implemented in the Ilastik software framework [21]. Two different parts were segmented based on the pixel-level classification: (1) Low-density macro pores and nano pores (filled with air) and (2) high-density photonic glass (see Fig. 2 in [1] for structural features description).

**Fig. 3:**
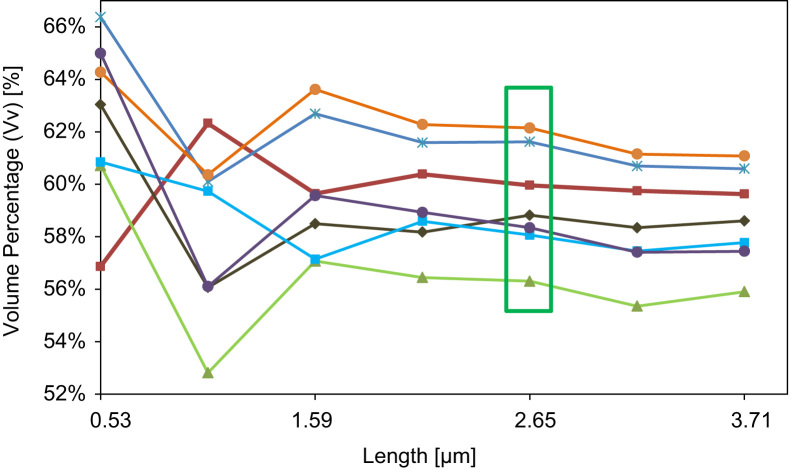
Example of representative volume of interest’ (RVI) test, based on the percentage volume (Vv) as a function of the edge length of a cubic volume of interest (VOI). Each line represents a growing VOI from a different sample region. The optimal VOI-size is indicated by the square marker and corresponds to a cube with a 2.65 µm edge (volume of 18.6 µm³).

**Fig. 4:**
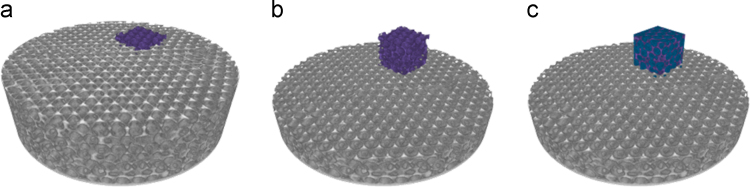
3D rendering of the PXCT tomogram from the mullite inverse opal photonic crystal before heat treatment showing an example of one analyzed volume of interest (VOI). A total of 10 cubic VOIs with an edge of 2.65 µm was analyzed per sample. Perpendicular cuts showing (b) the inverse opal photonic crystal phase in purple and (c) macro pores highlighted in dark blue. Sample diameter is 14.2 µm. (For interpretation of the references to color in this figure, the reader is referred to the web version of this article).

A test for finding the Representative Volume of Interest (RVI-test) was performed by plotting the Volume Percentage (Vol. Perc.) of the void part (macro and nano pores) as a function of the volume of interest (VOI)-size, as shown in Fig. 3. The representative VOI-size is found when the observed Vol.Perc. of all the considered VOIs are approaching the same value and when the variation of the Vol.Perc. is considered stable, where the quantified values can be said to no longer be effected by the varying size of the considered VOI [22]. The RVI-test resulted in a representative cubic VOI with an edge size of 2.65 µm (18.6 µm³ of total volume). The quantitative analysis of the sample features was performed on the considered VOIs (10 per sample, example in Fig. 4) using the Pore3D software library [19], from which a set of quantitative parameters were extracted, further described in the following paragraphs.

**Fig. 5:**
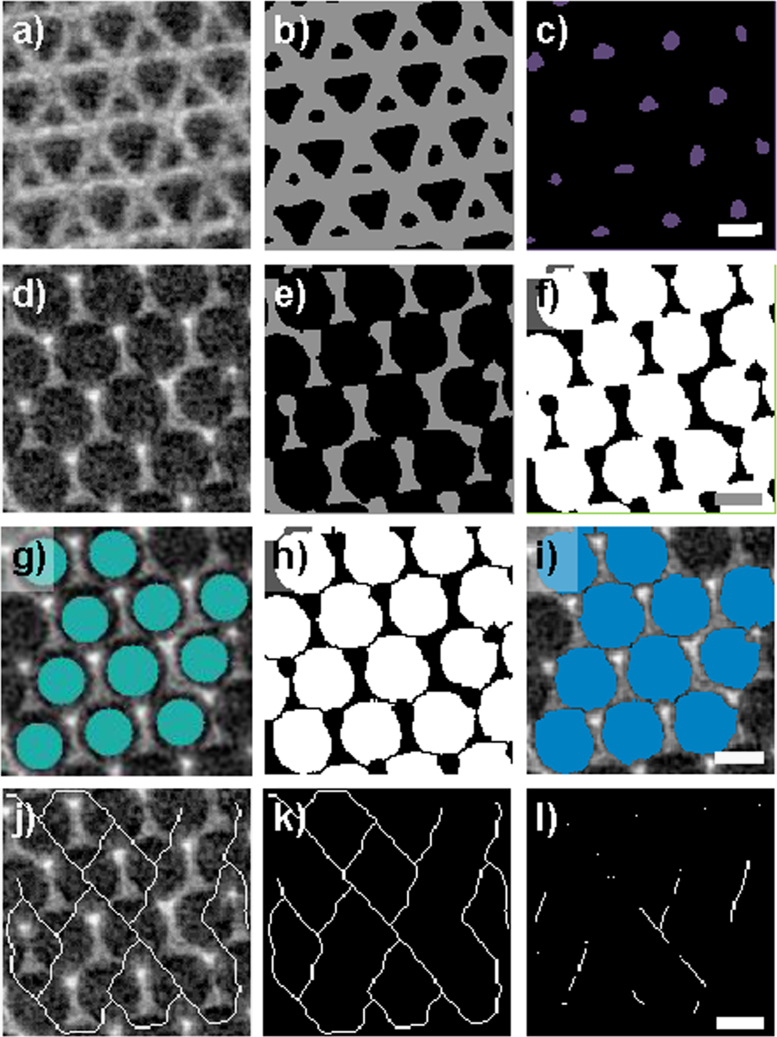
Two-dimensional slices obtained at different heights from the 3D volume of the mullite inverse opal photonic crystal highlighting the variety of structural features analyzed. (a and d) Show raw data, (b and e) show binary data showing pores in black and inverse opal photonic crystal phase in dark grey, and (b and f) show macro pores at different positions inside the sample (c) nano pores (g) inscribed blobs inside the macro pores sites; the inscribed blobs stopped growing as soon as they reach a wall (in 3D), which resulted in an absolute diameter value smaller than that of the real watershedded pores; (h and i) watershedded pores were region grown using inscribed blobs as ‘seeding points’ (in order to remove half-pores at the borders). Computed image skeleton in (j and k) 2D (l) 3D; the skeletonizing algorithm calculates the closest path to the next neighboring feature, which is often not located in the same 2D plan, since a 3D structure is considered. Regions in which the connection points are not clear pictured (compare d and e) are considered as walls by the skeletonizing algorithm. Scale bars, 500 nm.

**Fig. 6:**
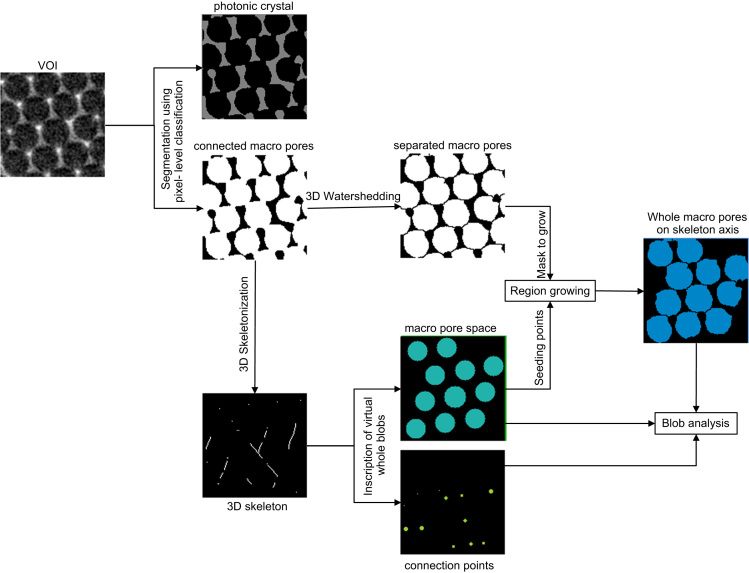
Schematic drawing of the applied image processing protocol in order to segment whole macro pores, as well as inscription of blobs in the macro pore space and at the connection points (necks). All image processing steps were performed in 3D. The results of these steps were used for the 3D quantification. 2D images are used in the schematic drawing with the sole purpose of easing the visualization of the inverse photonic crystal features.

**Fig. 7:**
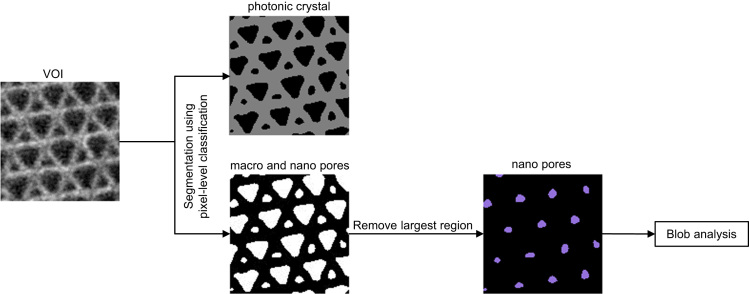
Schematic drawing of the applied image processing protocol in order to segment the nano pores. Since all the macro pores are connected with each other, the ‘remove largest region’ algorithm successfully removes this region. All image processing steps were performed in 3D. The results of these steps were used for the 3D quantification. 2D images are used in the schematic drawing with the sole purpose of easing the visualization of the inverse photonic crystal features.

An image skeleton representing the shortest path for interconnected pores was computed using the Gradient Vector Flow (GVF) algorithm [23] using the following parameters: Scale = 1.00, hierarchy = 0.40, and connectivity = 26. The image skeleton was further used to calculate a number for the connectivity density (*β*) in µm^−3^ of the pores, according to Eq. (1).

(1) $$\beta =\frac{\left(1-\chi_{v}\right)}{V}$$

where the Euler number (*χ*_v_) is defined as the difference between the number of nodes and branches (*χ*_V_=(no.nodes − no.branches)) along the extracted image skeleton and where *V* is the volume (in µm^3^) of the given VOI. Only the longest connected skeleton was considered for the analysis, while non-connected branches were filtered out. Inside the middle of the pore space and at the branches of the image skeleton, that is, at the passage through the connection points of the pores, virtual spheres or ellipsoids (hereinafter referred to as blobs) were inscribed and their radiuses were allowed to increase until they touched the inner border of the macro pore or the border of the connection points (neck in-between the macro pores). Hereinafter, so called ‘Blob Analysis’ was used to compute the diameter of the inscribed blobs, which served as an estimate of the mean diameter of the macro pore space and the opening diameter of the connection points. In another step, the macro pores were separated from each other using a ‘Distance Transform Watershed 3D’ algorithm in the MorphLibJ package [24] implemented in ImageJ-Fiji using the following parameters: Borgefors (3,4,5) and normalization dynamic = 1. Hereinafter, only macro pores laying on the longest connected skeleton axis were selected via region growing, by utilizing the inscribed blobs at the macro pores as ‘seeding points’. Finally the macro pore size was computed using ‘Blob analysis’, thereby, only considering watershed-separated whole macro pores laying on the skeleton axis, thus excluding both cut macro pores or macro pores not connected to the longest image skeleton (see Figs. 5 and 9). Furthermore, the size of nano pores was computed directly using ‘Blob Analysis’, following a separation of the nano pores from the normal macro pores using the ‘Remove Largest Region’ algorithm in the MorphLibJ package [24] implemented in ImageJ-Fiji. Schematic drawings of the image processing protocols described above are visualized in Figs. 6 and 7.

### 2.5 Macro pores 3D quantification

**Fig. 8:**
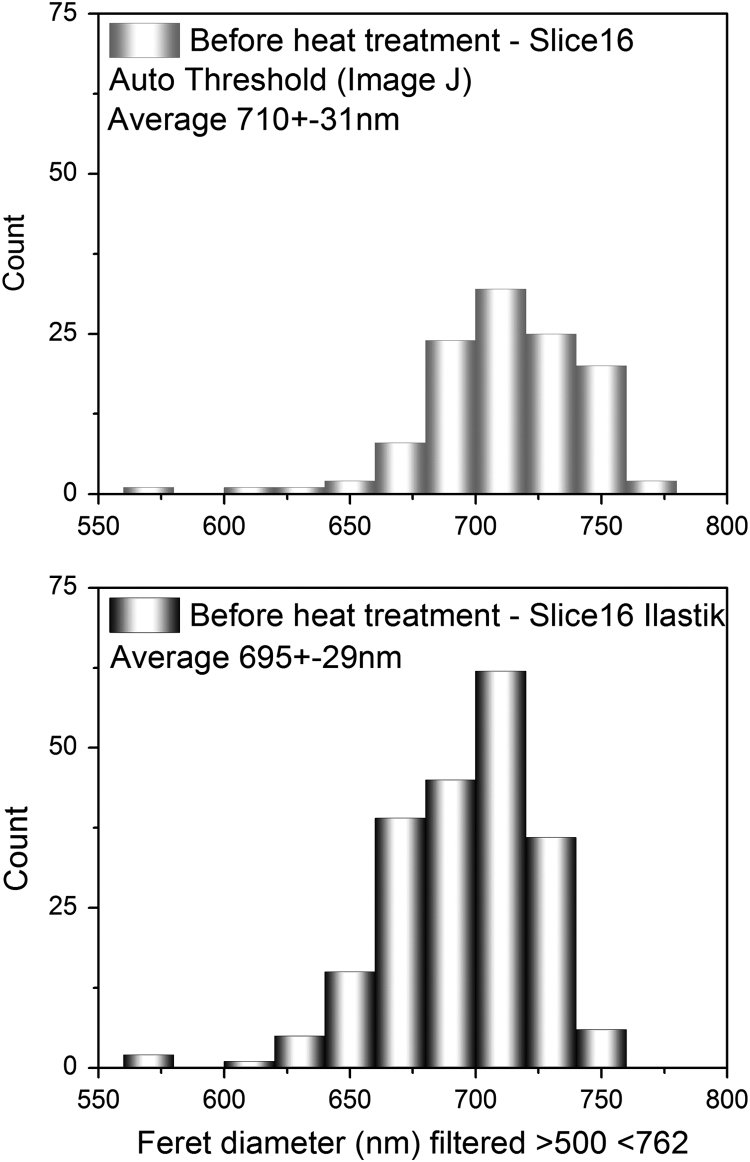
Size distribution of macro pores in a 2D-analysis of a selected slice from the PXCT data of the sample before the heat treatment, according to two different threshold methods.

As mentioned in the section ‘3D Structural changes analysis by high-resolution ptychography’ in [1], the interpretation of the 3D quantitative analysis performed on the PXCT dataset, could include a possible contribution from the volume between two non-overlapping pores (if at some points the inverse opal photonic crystal phase is smaller than the slice thickness), which is referred to as ‘partial volume effect’ [25]. Such an effect could impose challenges when segmenting the acquired data, especially if only standard grey-level thresholding methods are applied, which is why in this study a more advanced automated (supervised) pixel-level classification method (based on intensity, edge, and texture) was utilized. It is also important to point out that both phases (inverse photonic crystal and pores) have a distribution of gray scale values related to the Z-contrast and density contrast, which directly influences the signal during data collection. Furthermore, the macro pores are in general considered to be perfect spheres (following what is exposed in the literature data and the information from the PS spheres׳ supplier), which was not supported by our analysis. Deviations of the macro pore size in relation to the original PS value are reported even when different measurements techniques are employed [26], indicating that value fluctuations are acceptable. The first analysis of the macro pores size was performed by inscribing blobs followed by the ‘Blob analysis’ method (previously described), resulting in values with higher sphericity (near 1). It was noted then, that this method considers the growth of a either a spherical or ellipsoidal blob up to a point where this structure touches a ‘wall’ (in our case, the inverse opal photonic crystal shell), which for the algorithm is represented by a certain gray value defined by the hereby applied segmentation approach (compare Fig. 5d–i). As the ‘Blob analysis’ is performed in 3D, a mean value of the macro pore size will be obtained, which will by definition have a lower average diameter than that obtained by 2D-analysis of a selected slice (Fig. 8), regardless of the segmentation method used, since the 2D-analysis considers only the broadest possible diameter of each pore.

**Fig. 9:**
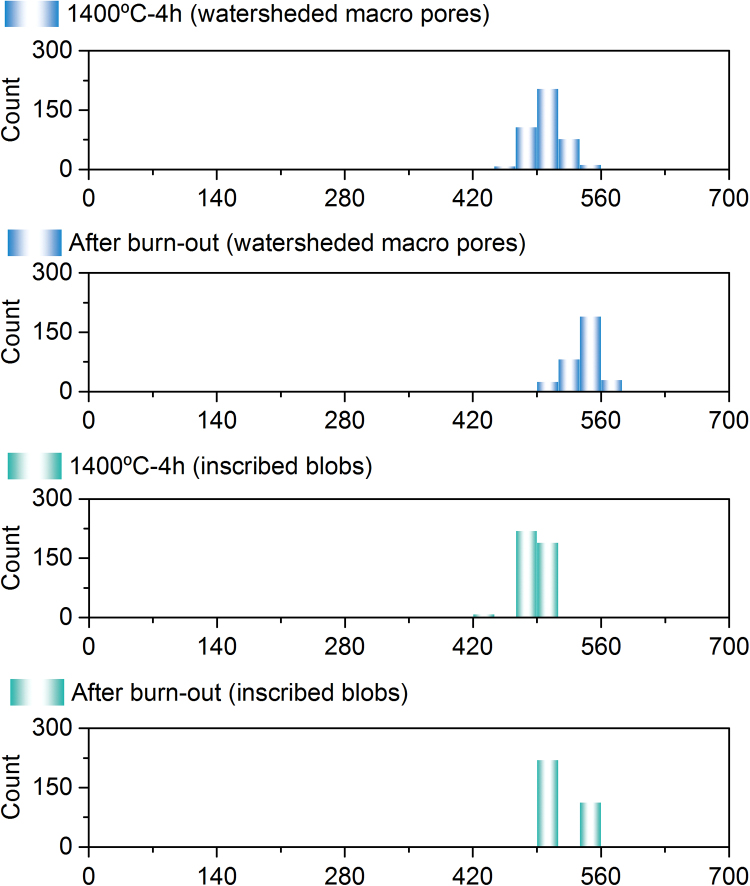
Size distribution of macro pores, according to the different analysis’ methods employed. Refer to Figs. 5 and 10 for the visualization of such features.

**Fig. 10:**
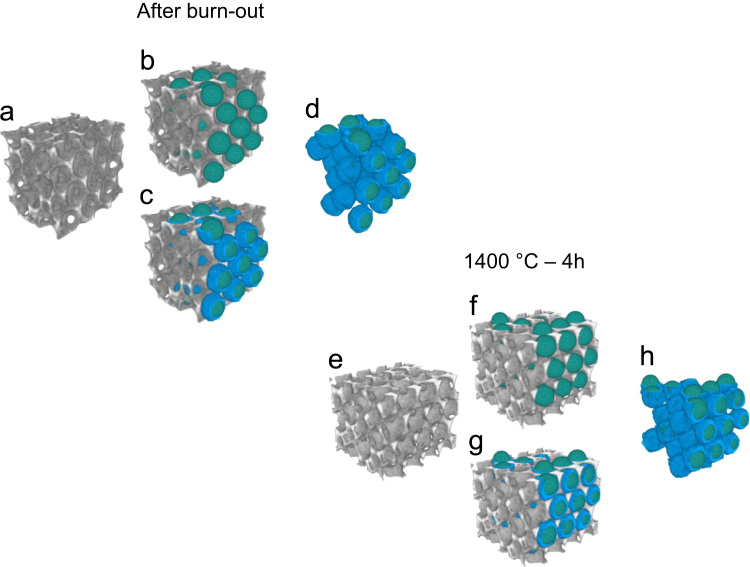
3D rendering of selected VOIs from the PXCT dataset of the mullite inverse photonic crystals (a–d) before and (e–h) after the heat treatment at 1400 °C for 4 h showing the macro pores (blue) and the inscribed blobs (green). Macro pores were analyzed via (b and f) inscribed blobs in green and (c and g) watershed-segmented pores in blue. A small perpendicular cut was applied to ease the visualization of the structural features. VOIs edge original size in (a and e) equals to 2.65 µm (volume of 18.6 µm^3^). (For interpretation of the references to color in this figure, the reader is referred to the web version of this article).

Although the border (half-pores) were excluded from the analysis, the larger interstitial sites could still be considered (theoretical calculated values based on [27] are between 170 and 315 nm, also considering a sphere). Alternatively, a different segmentation method was applied (Watershedding followed by region growing, utilizing the inscribed blobs as ‘seeding points’), which resulted in a larger quantified diameter of the considered macro pores (Fig. 9). The 3D rendering associated with these two different methods can be visualized in Fig. 10, where one can clearly see the differences concerning the macro pores diameters measured by both methods. Finally, the values discussed in this work consider a combination of these two methods.

## References

[1] Furlan K.P., Larsson E., Diaz A., Holler M., Krekeler T., Ritter M., Eich M., Schneider G.A., Blick R., Greving I., Zierold R., Janssen R. (2018). Photonic materials for high-temperature applications: synthesis and characterization by X-ray ptychographic tomography. Appl. Mater. Today..

[2] Gorham C.S., Gaskins J.T., Parsons G.N., Losego M.D., Hopkins P.E. (2014). Density dependence of the room temperature thermal conductivity of atomic layer deposition-grown amorphous alumina (Al_2_O_3_). Appl. Phys. Lett..

[3] Lee J.-H., Kim U.-J., Han C.-H., Rha S.-K., Lee W.-J., Park C.-O. (2004). Investigation of silicon oxide thin films prepared by atomic layer deposition using SiH_2_Cl_2_ and O_3_ as the precursors. Jpn. J. Appl. Phys..

[4] Wang H.L., Lin C.H., Hon M.H. (1997). The dependence of hardness on the density of amorphous alumina thin films by PECVD. Thin Solid Films.

[5] Furlan K.P., Pasquarelli R.M., Krekeler T., Ritter M., Zierold R., Nielsch K., Schneider G.A., Janssen R. (2017). Highly porous α-Al_2_O_3_ ceramics obtained by sintering atomic layer deposited inverse opals. Ceram. Int..

[6] Bachmann J., Zierold R., Chong Y.T., Hauert R., Sturm C., Schmidt-Grund R., Rheinlander B., Grundmann M., Gosele U., Nielsch K. (2008). A practical, self-catalytic, atomic layer deposition of silicon dioxide. Angew. Chem. Int. Ed..

[7] Han L., Chen Z. (2013). High-quality thin SiO_2_ films grown by atomic layer deposition using tris(dimethylamino)silane (TDMAS) and ozone. ECS J. Solid State Sci. Technol..

[8] Hirose F., Kinoshita Y., Shibuya S., Narita Y., Takahashi Y., Miya H., Hirahara K., Kimura Y., Niwano M. (2010). Atomic layer deposition of SiO_2_ from tris(dimethylamino)silane and ozone by using temperature-controlled water vapor treatment. Thin Solid Films.

[9] Hirose F., Kinoshita Y., Shibuya S., Narita Y., Miya H., Hirahara K., Kimura Y., Niwano M. (2009). Low-temperature-atomic-layer-deposition of SiO2 with tris(dimethylamino)silane (TDMAS) and ozone using temperature controlled water vapor treatment.

[10] Pradhan S.K., Tanyi E.K., Skuza J.R., Xiao B., Pradhan A.K. (2015). Electrical behavior of atomic layer deposited high quality SiO_2_ gate dielectric. J. Vac. Sci. Technol. A.

[11] Holler M., Raabe J., Wepf R., Shahmoradian S.H., Diaz A., Sarafimov B., Lachat T., Walther H., Vitins M. (2017). OMNY PIN—A versatile sample holder for tomographic measurements at room and cryogenic temperatures. Rev. Sci. Instrum..

[12] Holler M., Raabe J., Diaz A., Guizar-Sicairos M., Wepf R., Odstrcil M., Shaik F.R., Panneels V., Menzel A., Sarafimov B., Maag S., Wang X., Thominet V., Walther H., Lachat T., Vitins M., Bunk O. (2018). OMNY-A tomography nano cryo stage. Rev. Sci. Instrum..

[13] Huang X., Yan H., Harder R., Hwu Y., Robinson I.K., Chu Y.S. (2014). Optimization of overlap uniformness for ptychography. Opt. Express.

[14] Thibault P., Dierolf M., Menzel A., Bunk O., David C., Pfeiffer F. (2008). High-resolution scanning X-ray diffraction microscopy. Science.

[15] Thibault P., Guizar-Sicairos M. (2012). Maximum-likelihood refinement for coherent diffractive imaging. New J. Phys..

[16] Guizar-Sicairos M., Diaz A., Holler M., Lucas M.S., Menzel A., Wepf R.A., Bunk O. (2011). Phase tomography from X-ray coherent diffractive imaging projections. Opt. Express.

[17] Guizar-Sicairos M., Boon J.J., Mader K., Diaz A., Menzel A., Bunk O. (2015). Quantitative interior X-ray nanotomography by a hybrid imaging technique. Optica.

[18] Schindelin J., Arganda-Carreras I., Frise E., Kaynig V., Longair M., Pietzsch T., Preibisch S., Rueden C., Saalfeld S., Schmid B., Tinevez J.-Y., White D.J., Hartenstein V., Eliceiri K., Tomancak P., Cardona A. (2012). Fiji: an open-source platform for biological-image analysis. Nat. Methods.

[19] Brun F., Mancini L., Kasae P., Favretto S., Dreossi D., Tromba G. (2010). Pore3D: a software library for quantitative analysis of porous media. Nucl. Instrum. Methods Phys. Res. Sect. A.

[20] Ollion J., Cochennec J., Loll F., Escudé C., Boudier T. (2013). TANGO: a generic tool for high-throughput 3D image analysis for studying nuclear organization. Bioinforma. (Oxf., Engl.).

[21] C. Sommer, C. Straehle, U. Kothe, F.A. Hamprecht, Interactive learning and segmentation toolkit, in: Proceedings of the Eigth IEEE International Symposium., 2011.

[22] Bear J. (1988). Dynamics of fluids in porous media.

[23] Brun F., Dreossi D. (2010). Efficient curve-skeleton computation for the analysis of biomedical 3d images - biomed 2010. Biomed. Sci. Instrum..

[24] Legland D., Arganda-Carreras I., Andrey P. (2016). MorphoLibJ: integrated library and plugins for mathematical morphology with ImageJ. Bioinforma. (Oxf., Engl.).

[25] Barrett J.F., Keat N. (2004). Artifacts in CT: recognition and avoidance. Radio Graph..

[26] Goldenberg L.M., Wagner J., Stumpe J., Paulke B.-R., Görnitz E. (2002). Ordered arrays of large latex particles organized by vertical deposition. Langmuir.

[27] Wang J., Ahl S., Li Q., Kreiter M., Neumann T., Burkert K., Knoll W., Jonas U. (2008). Structural and optical characterization of 3D binary colloidal crystal and inverse opal films prepared by direct co-deposition. J. Mater. Chem..

