# Supplementary material for: Dataset of ptychographic X-ray computed tomography of inverse opal photonic crystals produced by atomic layer deposition
Source: Data Brief. 2018 Oct 27;21:1924–36. doi: 10.1016/j.dib.2018.10.076 (PMC6260412; doi:10.1016/j.dib.2018.10.076)
Supplement: Supplementary file 1 — Supplementary material. [file mmc1.docx]

**Conflict of Interest Form**

Declarations of interest: none.
